# Accuracy of two prognostic indexes to predict mortality in older adults with advanced dementia

**DOI:** 10.1590/1980-5764-DN-2021-0028

**Published:** 2022

**Authors:** Beatriz Noele Azevedo Lopes, Flavia Barreto Garcez, Claudia Kimie Suemoto, Lilian Schafirovits Morillo

**Affiliations:** 1 Universidade de São Paulo, Faculdade de Medicina, Divisão de Geriatria, São Paulo SP, Brazil.

**Keywords:** Aged, Dementia, Prognosis, Survival Analysis, Mortality, Idoso, Demência, Prognóstico, Análise de Sobrevida, Mortalidade

## Abstract

Dementia is a cause of disability among older adults. Accessing advanced dementia prognosis is a challenge.

**Objective:**

The objective of this study was to evaluate the accuracy of the Charlson and Carey indexes in predicting 3-year survival of older adults with advanced dementia.

**Methods:**

This is a retrospective cohort study of 238 patients aged ≥60 years with advanced dementia from an outpatient clinic and classified as stage ≥6A by using the Functional Assessment Staging scale. We excluded patients with missing data. We reviewed the semi-structured interview (clinical, sociodemographic, and functional data) from the baseline visit. This information was used to calculate 3-year mortality risks according to the Charlson and Carey indexes. We used Cox proportional hazard models to evaluate the associations of all-cause mortality with both indexes, adjusted for sociodemographic variables. We used Harrell’s C measure to determine the discrimination. We calculated the absolute differences between observed and predicted 3-year mortality risks for each index for calibration.

**Results:**

In 238 patients, the average age was 80.5±7.8 years, with 36% being men. The median follow-up time was 1.8 years (0.05–3.0). The 3-year all-cause mortality rate was 50% (119 deaths). The Carey index was associated with mortality, with one point increase related to a 15% increase in the mortality risk (hazard ratio [HR]=1.15, 95% confidence interval (95%CI) 1.06–1.25, p=0.001), even after adjustment. Accuracy for the Charlson index and Carey index was 0.55 (95%CI 0.49–0.60) and 0.60 (95%CI 0.52–0.62), respectively, with no difference between them (p=0.44).

**Conclusions:**

Both indexes had poor discrimination and calibration performances in predicting 3-year mortality in patients with advanced dementia.

## RESUMO.

Demência é uma causa de incapacidade e dependência em idosos. A avaliação prognóstica na fase avançada é desafiadora.

**Objetivo:**

Avaliar a acurácia dos índices de Charlson e Carey na predição de mortalidade em até três anos de idosos com demência avançada.

**Métodos:**

Estudo de coorte retrospectiva que incluiu 238 pacientes acompanhados em ambulatório especializado em um país de renda média, com idade ≥60 anos e demência avançada classificada como estágio ≥6A pela escala *Functional Assessment Staging* (FAST). Foram excluídos pacientes com dados incompletos para análise. Realizou-se revisão da primeira consulta, que consiste em entrevista com dados clínicos, sociodemográficos e funcionais utilizados para calcular a probabilidade de óbito em três anos, de acordo com os índices. Foram usados modelos de risco proporcional de Cox para avaliar as associações de mortalidade por todas as causas com os dois índices, ajustados para variáveis sociodemográficas. As discriminações dos dois modelos foram comparadas usando o cálculo C de Harrell. Para a calibração, foram calculadas as diferenças absolutas entre os riscos observados e preditos por cada um dos índices.

**Resultados:**

Foram avaliados 238 pacientes, com média de idade de 80,5±7,8 anos, 36% do sexo masculino. A mediana do tempo de acompanhamento foi de 1,8 anos (intervalo interquartil=0,05–3,0). A taxa de mortalidade por todas as causas em três anos foi de 50% (119 óbitos). O índice de Carey foi associado à mortalidade, mas o de Charlson não. Um aumento de 1 ponto no Carey foi relacionado a aumento de 15% no risco de morte (hazard ratio [HR]=1,15, intervalo de confiança [IC95%] 1,06–1,25, p=0,001), mesmo após ajuste para variáveis sociodemográficas. A acurácia do índice de Charlson foi de 0,55 (IC95% 0,49–0,60) e a do índice de Carey de 0,60 (IC95% 0,52–0,62), sem diferença significativa na discriminação (p=0,44). Ambos os índices tiveram *performances* insatisfatórias na discriminação e na calibração.

**Conclusões:**

O índice de Carey foi associado à mortalidade, porém esse resultado não foi encontrado para o índice de Charlson. Ambos os índices tiveram desempenho insatisfatório na discriminação e na calibração para predizer a mortalidade em três anos em pacientes com demência avançada, o que indica que esses escores não são recomendados para predizer a mortalidade nessa população.

## INTRODUCTION

In 2015, approximately 46.8 million people were living with dementia worldwide. This number is expected to double every 20 years, reaching 131.5 million in 2050^1^. Dementia is the most important and independent cause of disability and mortality in older people living in low- and middle-income countries (LMICs), and almost 66% of people with dementia live in these areas^2^. Alzheimer’s disease (AD), the most common etiology of dementia, is the sixth leading cause of death in the United States and the fifth cause of death in individuals aged 65 years or older globally, with a mortality rate similar to cerebrovascular disease^3,4^. People with dementia have an average mortality risk of 2.6 times higher than those in the same age group without dementia, and in LMIC, the mortality rates are 1.6–5.7 times higher when compared to people without dementia^2^.

Given that AD patients live many years in advanced stages of dementia after the diagnosis^5-7^, the identification of factors associated with adverse outcomes in this population could help the management of care and also the better allocation of healthcare resources^7^. Although dementia is an independent predictor of decreased survival, it is often not recognized as a terminal disease, even in advanced stages^8,9^. One possible explanation relies on the challenge of accurately estimating life expectancy in this stage, which becomes a barrier for high-quality end-of-life care to these patients. Previous studies have tried to develop and validate scores to estimate prognosis in advanced dementia, and most of them restricted to nursing home residents and predictions of 6-month mortality but failed to reach adequate accuracy or generalizability^10–12^. These unsatisfactory results were summarized in a systematic review, suggesting the absence of an ideal scale to measure dementia severity and predict 6-month mortality^13^.

Considering the lack of agreement in previous studies and the fact that severe stages of dementia can last more than 6 months^5,7^, we searched for validated mortality prediction scores to explore their performance in patients with advanced dementia. The Charlson comorbidity index (CCI) is a widely used risk score that estimates mortality risk based on the presence of comorbidities^14^. The CCI has good performance in predicting survival in 1–10 years and is an independent predictor of short- and long-term mortality in elderly populations^15,16^. Another systematic review presented several geriatric prognostic indexes designed for older adults who did not have a specific terminal disease^17^. Among the various tools, the Carey prognostic index is a multidimensional index that predicts 3-year mortality risk among frail older adults living in the community. In this score, disability is a strong independent predictor of mortality^18^.

Although many geriatric prognostic indexes were developed, there is no accurate index to predict mortality in older adults with advanced dementia. Knowing the importance of estimating survival to provide better care options to these patients, which includes an appropriate level of palliation^6,13^, our study aimed to evaluate the accuracy of the CCI and Carey prognostic indexes in predicting 3-year survival in community-dwelling older adults with advanced dementia. We hypothesized that the Carey prognostic index would predict all-cause mortality in patients with advanced dementia, and it would have a better performance than the CCI since the Carey index includes functional variables.

## METHODS

### Participants

This retrospective cohort study included 358 patients who were admitted to an advanced dementia outpatient clinic since 2008, located in a university hospital of an upper-middle-income country (São Paulo, Brazil). The inclusion criterion was individuals aged 60 years and older with advanced dementia who were classified as stage 6A or higher by the Functional Assessment Staging (FAST) scale^19^. Patients needed to have at least one outpatient visit before death to be enrolled. We tried to contact participants who were lost during the follow-up (last visit 6 months or more, or discharge to another service) by phone calls, or we retrieved the outcome information of these patients from the hospital electronic medical record system. The exclusion criteria were patients with missing medical records. The follow-up time in years was calculated from the first outpatient evaluation until death or the last visit date. The review of the medical records was carried out between October 2017 and July 2018. The local ethics committee approved this study (protocol number 2.427.160). Before enrollment, informed consent was obtained from the participants’ relatives.

### Clinical evaluation

A single researcher reviewed the first outpatient visit from medical records, which contains a semi-structured interview with demographics and functional and clinical data. Demographic information included age, sex, years of education, and race. Functional status was based on Alzheimer’s Disease Cooperative Study Activities of Daily Living Inventory for Severe Alzheimer’s Disease (ADCS-ADL-severe)^20^. This scale assessed the ability of patients with moderate-to-severe dementia to perform ADL and was already adapted to the Brazilian population (unpublished data). When ADCS-ADL-severe was not available, this information was retrieved from the medical interview. The evaluated ADLs were toileting and dressing, the same ones used in the Carey index. Dependence in toileting was considered if the patient scored less than 3 points in this task on the ADCS-ADL-severe. The patients were considered partially dependent on dressing if they scored 3 or fewer points in this task and fully dependent if they scored zero. Regarding cognitive evaluation, the Mini-Mental State Examination (MMSE) and the Severe Mini-Mental State Examination (SMMSE) were performed^21,22^. The SMMSE was designed to briefly assess cognitive domains relatively preserved in moderate-to-severe AD^22^ and included simpler commands and questions related to autobiographical knowledge, constructional praxis, phonological loop, semantic verbal fluency, and receptive and expressive language skills, along with elementary executive functions and visual-spatial abilities, which are likely to be preserved in severely impaired patients^23^. This score ranges from 0 to 30 points and has already been adapted to the Brazilian Portuguese language^24^. Significant associations were observed between the SMMSE and other functional scales and this instrument correlated with MMSE in patients who had a Mini-Mental score of fewer than 10 points^25^.

### Comorbidity evaluation

The CCI was originally developed to predict the long-term survival of individuals with cancer by assigning weights to specific diseases^14^. Participants receive 1 point for each of the following diseases: myocardial infarction, heart failure, peripheral vascular disease, cerebrovascular disease, dementia, chronic pulmonary disease, connective tissue disease, ulcer disease, mild liver disease, and diabetes. Leukemia, lymphoma, hemiplegia, any tumor, diabetes with end-organ damage, and moderate or severe renal disease scored 2 points each. Moderate or severe liver disease scored 3 points, whereas metastatic solid tumors and AIDS scored 6 points each. Moderate/severe renal disease includes patients on dialysis, those who had a transplant, and those with uremia or serum creatinine >3 mg/dL. Solid tumors were those without documented metastases, excluding non-melanoma skin cancer. The total score is calculated by adding the points for each disease.

The Carey index was developed to accurately stratify community-dwelling frail older adults into groups according to their risk of mortality. This model assigned points to specific sociodemographic, clinical, and functional variables: male sex (2 points), age (75–79 years: 2 points; 80–84 years: 2 points; ≥85 years: 3 points), dependence in toileting (1 point), dependence in dressing (partial dependence: 1 point; full dependence: 3 points), cancer (2 points), heart failure (3 points), chronic obstructive pulmonary disease (1 point), and renal insufficiency or failure (3 points). The total score is calculated by adding the points for each item. Patients were divided into three groups according to their scores. Some variables were defined by clinician judgment rather than a strict definition^18^. For example, there was no specific creatinine clearance defining renal failure in the Carey study; thus, we used creatinine clearance below 45 mL/min by the Chronic Kidney Disease Epidemiology Collaboration (CKD-EPI) formula to classify renal insufficiency or failure^26^. In addition, information was not available about illness severity^18^.

### Statistical analysis

We compared participants who died during the follow-up with those who survived using χ^2^ and Fisher’s exact tests for categorical variables and using unpaired Student’s *t*-test for continuous variables. The outcome was time to death during a 3-year follow-up in older adults with advanced dementia after admission to the advanced dementia outpatient clinic. We used Cox proportional hazard models to evaluate the associations of all-cause mortality with the Charlson and Carey indexes adjusted for sociodemographic variables. Then, we evaluated the accuracy of Charlson and Carey prognostic indexes as predictors of mortality during this period. Calibration for both indexes was evaluated by comparing the predicted and observed risk of death by quartiles of predicted risk. We calculated the absolute differences between observed and predicted 3-year mortality risks within each quartile of predicted risk and considered differences in risk of <10% as an indication of acceptable calibration^17^. We also fitted a linear regression model to compare the predicted and observed 3-year mortality risk for the Charlson and Carey indexes. Good calibration is present when the slope is close to 1.0^27^.

We used Harrell’s C measure to determine the discrimination of the two models since data were analyzed using survival analysis^28^. We compared the predictive power of the CCI and the Carey index using the *lincom* function, which calculates the confidence intervals (CIs) and p-values for the difference of Harrell’s Cs between the two indexes^29^. We used Stata version 15 (StataCorp, TX, USA) for statistical analyses. The alpha level was set at 0.05 in the two-tailed tests.

## RESULTS

**Figure 1:**
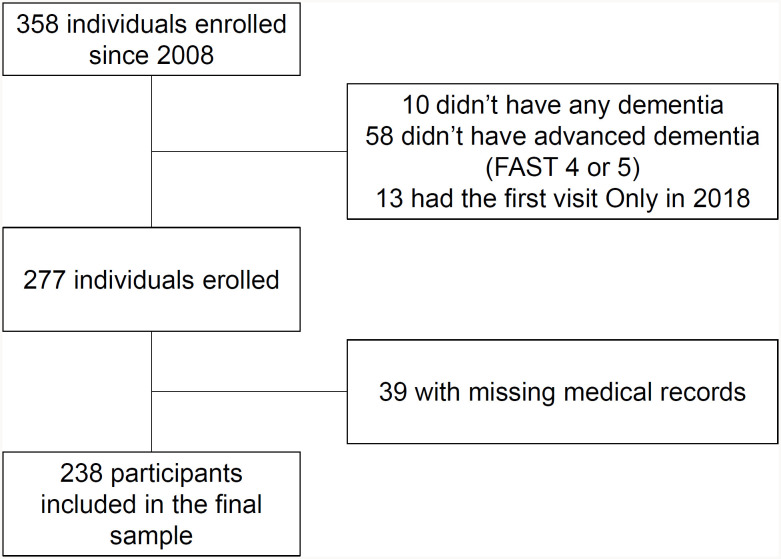
Flowchart of study participants.

**Figure 2:**
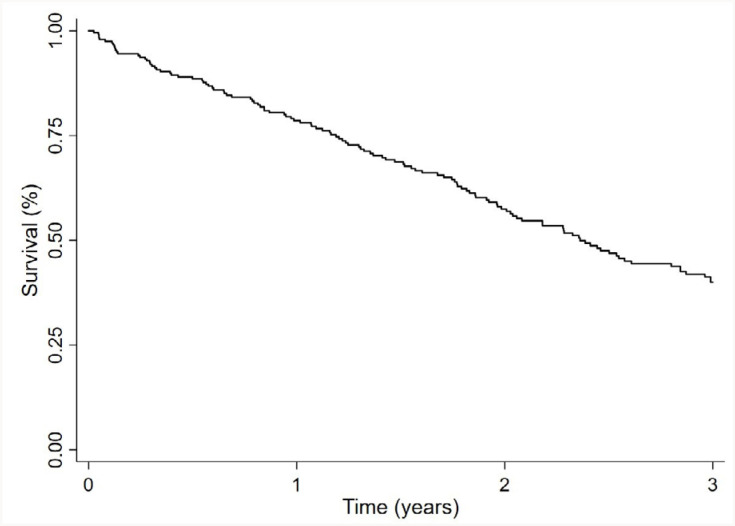
Kaplan-Meier survival curve during the 3 years of follow-up.

From 2008 to 2018, 358 individuals were admitted to the advanced dementia outpatient clinic. After applying the exclusion criteria, 238 patients were included in the final study sample, as shown in the flowchart of patient selection and exclusion criteria (Figure 1). The mean age was 80.5±7.8 years old, and 36% were men. Most patients (67.6%) were classified as moderate or severe dementia by the FAST scale (stage 6). The median follow-up time was 1.8 years (interquartile range=0.05–3.0). The mean scores on the MMSE and SMMSE were 5.9±5.5 and 12.7±8.8 points, respectively. The most common comorbid conditions were hypertension (68%), cerebrovascular disease (29%), diabetes (22%), heart failure (17%), and myocardial infarct (13%). Moderate/severe renal disease was present in 16% of patients and was associated with mortality. Male gender was the only sociodemographic variable associated with mortality. The functional parameter dependence in toileting was borderline associated with mortality risk (Table 1). The mean CCI score in this cohort was 2.5±1.6 points, and the mean Carey index was 7.4±2.5 points. The points distribution among the participants were different for the CCI and Carey index, as the majority of the patients had lower scores in the CCI (60% scored 1 or 2 points) and higher scores in the Carey index (62% scored 6–9 points). The frequency of 3-year all-cause mortality was 50% (n=119), and 29 participants had a single outpatient visit before death. The Kaplan–Meier survival curve is presented in Figure 2.

**Table 1:** Baseline characteristics of study participants (n=238).

		Total (n=238)	Survivors (n=119)	Dead (n=119)	p-value
Sociodemographic					
	Age (years), mean (SD)*	80.5 (7.8)	80.2 (7.9)	80.8 (7.8)	0.59
	Male, n (%)†	86 (36.1)	34 (28.6)	52 (43.7)	0.01
	Education (years), mean (SD)*	4.4 (4.1)	4.1 (3.9)	4.7 (4.4)	0.33
Race, n (%)†					0.59
	White	174 (73.4)	88 (74.6)	86 (72.3)	
	Brown	33 (13.9)	18 (15.2)	15 (12.6)	
	Black	22 (9.3)	8 (6.8)	14 (11.8)	
	Asian	8 (3.4)	4 (3.4)	4 (3.4)	
Comorbidities					
	Hypertension, n (%)†	162 (68.1)	84 (70.6)	78 (65.5)	0.40
	Cerebrovascular disease, n (%)†	70 (29.4)	34 (28.6)	36 (30.3)	0.78
	Diabetes, n (%)†	52 (21.9)	27 (22.7)	25 (21.0)	0.75
	Diabetes with end-organ damage, n (%)§	12 (5.0)	7 (5.9)	5 (4.2)	0.55
	Heart failure, n (%)†	40 (16.8)	16 (13.4)	24 (20.2)	0.17
	Moderate/severe renal disease, n (%)†	38 (16.0)	13 (10.9)	25 (21.0)	0.03
	Myocardial infarct, n (%)†	30 (12.6)	13 (10.9)	17 (14.3)	0.43
	Cancer, n (%)†	28 (11.8)	12 (10.1)	16 (13.4)	0.42
	Metastatic solid tumor, n (%)§	1 (0.4)	0 (0.0)	1 (0.8)	0.31
	Hemiplegia, n (%)§	20 (8.4)	11 (9.2)	9 (7.6)	0.64
	Chronic pulmonary disease, n (%)§	14 (5.9)	5 (4.2)	9 (7.6)	0.27
	Peripheral vascular disease, n (%)§	11 (4.6)	4 (3.4)	7 (5.9)	0.35
	Ulcer disease, n (%)§	7 (2.9)	2 (1.7)	5 (4.2)	0.25
	Connective tissue disease, n (%)§	1 (0.4)	0 (0.0)	1 (0.8)	0.32
	Leukemia, n (%)§	2 (0.8)	0 (0.0)	2 (1.7)	0.16
	Lymphoma, n (%)§	0 (0.0)	0 (0.0)	0 (0.0)	1.00
	Mild liver disease, n (%)§	3 (1.3)	0 (0.0)	3 (2.5)	0.08
	Moderate or severe liver disease, n (%)§	0 (0.0)	0 (0.0)	0 (0.0)	1.00
	AIDS, n (%)§	0 (0.0)	0 (0.0)	0 (0.0)	1.00
Functional status					
Dependence in toileting, n (%)§		210 (88.6)	100 (84.8)	110 (92.4)	0.06
Partial dependence on dressing, n (%)§		31 (13.1)	17 (14.4)	14 (11.8)	0.55
Full dependence on dressing, n (%)§		194 (81.9)	92 (78.0)	102 (85.7)	0.12

* Unpaired Student’s *t*-test;

† χ^2^ test

§ Fisher’s exact test.

The Carey index was associated with mortality, while the CCI was not (Table 2). One point increase in the Carey index was related to a 15% increase in the mortality risk (hazard ratio [HR]=1.15, 95%CI 1.06–1.25, p=0.001), even after adjustment for sociodemographic variables. Regarding the discrimination of the CCI to predict 3-year mortality in older adults with advanced dementia, Harrell’s C was 0.55 (95%CI 0.49–0.60). For the Carey index, Harrell’s C was 0.60 (95%CI 0.52–0.62) (Table 2). There was no significant difference in discrimination between the two indexes (p=0.44). For the calibration, the difference between the predicted and the observed 3-year mortality was less than 10% only in the third quartile for the CCI. The Carey index had an unsatisfactory calibration in all quartiles (Table 3).

**Table 2:** Association of the Charlson comorbidity index and the Carey index with all-cause mortality (n=238).

Index	Crude	p-value	Adjusted*	p-value
	Hazard ratio (95%CI)		Hazard ratio (95%CI)	
Carey	1.12 (1.04–1.20)	0.001	1.15 (1.06–1.25)	0.001
Charlson	1.09 (0.98–1.20)	0.108	1.07 (0.96–1.19)	0.22

* Cox proportional hazard regression models adjusted for age, sex, education, and race; 95%CI: 95% confidence interval.

**Table 3:** Differences between observed and predicted 3-year mortality risks within each quartile of predicted risk of the Charlson comorbidity index and the Carey prognostic index.

	Charlson comorbidity index			Carey prognostic index		
Quartiles*	Obs (%)†	Pred (%)§	Dif (%)||	Obs (%)†	Pred (%)§	Dif (%)||
4	75.0	105.6	30.6	52.5	150.1	97.6
1	56.3	25.8	-30.4	51.9	7.3	-44.5
2	60.9	46.3	-14.6	66.7	46.7	-19.9
3	65.8	66.7	0.9	70.0	83.1	13.1

* Total score points divided in four quartiles. For the CCI: 1 point (first quartile), 2 points (second quartile), 3 points (third quartile), and >3 points (fourth quartile). For the Carey index: 0–6 points (first quartile), 7 points (second quartile), 8–9 points (third quartile), and >9 points (fourth quartile).

† Percentage of observed 3-year mortality risks within each quartile.

§ Percentage of predicted 3-year mortality risks within each quartile.

|| Absolute differences between observed and predicted 3-year mortality risks within each quartile of predicted risk.

## DISCUSSION

We evaluated the accuracy of two prognostic indexes in predicting 3-year all-cause mortality in older adults with advanced dementia, who were followed for a median time of 1.8 years. The Carey index was related to mortality, with 1 point increase related to a 15% increase in the risk of death. Both the CCI and Carey prognostic index did not show good accuracy in estimating 3-year mortality in a sample of patients with advanced dementia. Although the Carey index had slightly better discrimination than CCI (Harrell’s C=0.60 vs. 0.55), the difference between Harrell’s C values was not significant. The discrimination found in this study was similar to other studies in advanced dementia^11,12^. Both indexes had unsatisfactory calibration. Nonetheless, we found that male gender and renal insufficiency were associated with 3-year mortality in this population, and dependence in toileting was the functional variable that came closest to the association with mortality.

Our population consisted mostly of frail community-dwelling older adults with multimorbidity. There was a high frequency of octogenarian participants (63%) in our study, and most of them were women (64%). Cardiovascular diseases were the most frequent comorbidity in our sample. Interestingly, most comorbidities were not related to mortality, except for renal insufficiency that had a significant relationship with increased 3-year mortality, especially when creatinine clearance was 45 mL/min or less. Previous investigations attempted to identify factors associated with survival in advanced dementia. Mitchell et al. developed and validated the Advanced Dementia Prognostic Tool (ADEPT), a score with 12 items related to poor survival (such as recent admission in a nursing home, age, male sex, shortness of breath, pressure ulcers, ADL score, bedfast, insufficient oral intake, bowel incontinence, body mass index [BMI] <18.5 kg/m^2^, weight loss, and congestive heart failure). This model had moderate accuracy and greater discrimination in estimating 6-month mortality compared with hospice eligibility guidelines^11^. Other studies found an association of age, male gender, cardiovascular disease, and diabetes with worse prognosis in patients with advanced dementia, demonstrating functional impairment as a relevant predictive factor in this condition.^10,12^ It is important to note that these studies were designed to predict only 6-month survival in advanced dementia^10–12^ or evaluate the prognosis only in nursing home patients^9–12^.

The grading scale most used to determine prognosis in end-of-life dementia is the FAST, which rates the severity of AD^3,19^. This instrument has limitation because it assumes stepwise disease progression. However, dementia is known to have a varying and multifaceted disease course, which limits the prognostic capacity of the FAST scale. Therefore, the data do not yet support its use for direct determinations of prognosis, but FAST can function as a criterion within a larger prognostic scale^3^. The Clinical Dementia Rating (CDR) applies to a wide range of mild to more severe stages of dementia, and it is useful when a global assessment of cognitive function is required^30^. Some studies showed that global CDR was an important mortality predictor^31,32^. However, these studies did not have patients with advanced dementia as their target population. One study evaluated patients with AD, but only 7% had severe dementia^31^. Another study included patients with early-onset AD, who had 5-year mortality two to three times higher than the general population^32^. ­Therefore, these results cannot be generalized to our population, since the early onset of symptoms with a mean age of 58 years is not representative of the late-onset dementia population^32^; and other dementia types were excluded, with fewer participants categorized in the advanced stage^31^. A recent study found that older age, male gender, higher dementia severity evaluated in three distinct dimensions (i.e., cognitive, behavioral, and clinical), undernutrition, and higher number of physical impairments predicted higher mortality risk in people with dementia, including in those with advanced dementia, although severe dementia represented only 8% of the sample^2^.

Differences in social support after the diagnosis of dementia, the timing of diagnosis, and the prevalence of medical comorbidities may explain why mortality is difficult to predict in this population^4^. Variability also exists in the survival for persons with specific dementia subtypes, as shown in a large Swedish cohort study that compared mortality ratios by dementia subtype with AD. The lowest mortality rate was found for mixed dementia (HR=1.32; 95%CI 1.22–1.44), followed by Parkinson’s disease dementia (HR=1.47; 95%CI 1.17–1.84), vascular dementia (HR=1.55; 95%CI 1.42–1.69), and dementia with Lewy bodies (HR=1.64; 95%CI 1.39–1.95). Frontotemporal dementia (HR=1.91, 95%CI 1.52–2.39) showed the highest mortality risk and the most rapid decline^33^. The association between dementia subtypes and mortality is controversial. In a recent study conducted in LMICs, dementia subtypes did not predict mortality in a dementia population sample^2^.

It is important to emphasize that both indexes evaluated in this study were not specifically designed to assess prognosis in patients with advanced dementia, which may also have influenced the results. CCI had a poor performance in our sample compared to a previous study^34^. Despite its wide use, the CCI was developed when some chronic diseases, such as AIDS, did not yet have available treatment. Therefore, CCI limitations are related to comorbidities’ classification and severity. For example, moderate/severe renal disease is only classified when the serum creatinine is above 3.0 mg/dL, which can reduce the sensitivity in detecting moderate renal dysfunction in older patients. Furthermore, the CCI was validated in a population with a low incidence of comorbidities, which can limit CCI use in dementia patients, who usually have a greater number of associated comorbidities^14^.

The Carey prognostic index was associated with mortality. The original study population was similar to ours, which consisted of frail community-dwelling older adults with multiple comorbidities, including dementia^18^. Moreover, the Carey index included functional parameters that have been associated with mortality in other studies^2,11^. Even so, the Carey index had unsatisfactory discrimination and calibration in our population. Regarding the Carey index limitations, in the original study, the presence of comorbid illnesses was based on clinician judgment rather than a strict definition (e.g., there was no specific creatinine clearance defining renal failure) and information was not available about the severity of illness. Also, the study population survived longer probably because of access to a broad range of services and consistent care across settings^18^.

Other studies demonstrated that mortality was associated with other factors related to complications of the advanced stage of dementia, like pneumonia, electrolyte abnormalities, reduction in nutrient intake >25% (due to dysphagia), and the presence of severe pressure injuries (due to immobility syndrome)^35^. Both CCI and Carey index do not include dementia-specific predictors found in these studies^2,35^. Based on these data, an index that includes functional and physical impairment, clinical and behavioral complications of severe stages, and quality of care should be considered in future studies of advanced dementia prognostication.

Our study has some limitations. It was a retrospective analysis with a small sample of patients from an advanced cognitive impairment outpatient clinic in a tertiary hospital. This selection bias might affect the generalizability of our results since all patients and caregivers received well-guided instructions from a specialized medical and gerontological team in the care of patients with advanced dementia. We lost 14% of participants because of incomplete data. Unfortunately, we could not perform multiple imputations because we did not have the relevant sociodemographic and clinical information to apply in this method. We did not use dementia etiology as a potential predictor, and functionality was not extracted based on a single criterion since when ADCS-ADL severe score was not available, this information was collected from the medical interview.

Regarding the strengths of this study, we tested two well-known prognostic scores of easy applicability, and we evaluated prognostic factors in various dementia etiologies, not only AD, as in most other studies. We included functional impairment criteria, an important mortality predictor in advanced dementia, in previous studies^2,10–12^. Finally, our study population was mostly community-dwelling older persons, different from previous investigations, which included only nursing home residents^10–12^. Therefore, our study contributed as a preliminary basis for the construction of a specific prognostic score for advanced dementia.

The Carey index was associated with mortality, while the CCI was not. In this study, both calibration and discrimination for the two indexes were not satisfactory, indicating that these indexes are not recommended for mortality estimation in individuals with advanced dementia. We found that male gender and renal insufficiency were predictors in the same population. Since we lack a unifying guideline for dementia prognostication, clinicians should plan and provide the best care to patients with advanced dementia guided by previous experiences and information regarding the disease stage and always consider the family’s and patients’ preferences. It is important to recognize the challenge of estimating prognosis in this population, and this study encourages further research on this topic.
